# Global trends and gaps in research related to latent tuberculosis infection

**DOI:** 10.1186/s12889-020-8419-0

**Published:** 2020-03-18

**Authors:** Liling Chaw, Lung-Chang Chien, Justin Wong, Ken Takahashi, David Koh, Ro-Ting Lin

**Affiliations:** 1 PAPRSB Institute of Health Sciences, Universiti Brunei Darussalam, Jalan Tungku Link BE1410, Gadong, Bandar Seri Begawan, Brunei Darussalam; 2 Epidemiology and Biostatistics, Department of Environmental and Occupational Health, School of Public Health, University of Nevada, Las Vegas, Las Vegas, NV 89119 USA; 3 Disease Control Division, Ministry of Health, Brunei Darussalam; Commonwealth Drive, BB3910, Bandar Seri Begawan, Brunei Darussalam; 4 Asbestos Diseases Research Institute, Concord, NSW 2139 Australia; 5 Saw Swee Hock School of Public Health, National University of Singapore, Singapore, 117549 Republic of Singapore; 6 Department of Occupational Safety and Health, College of Public Health, China Medical University, Room 1610, No. 91, Hsueh-Shih Road, Taichung, 40402 Taiwan

**Keywords:** Tuberculosis, Latent tuberculosis infection, Public health, Prevention, Research governance, Bibliometric analysis

## Abstract

**Background:**

There is a global commitment to eliminating tuberculosis (TB). It is critical to detect and treat cases of latent TB infection (LTBI), the reservoir of new TB cases. Our study assesses trends in publication of LTBI-related research.

**Methods:**

We used the keywords (“latent tuberculosis” OR “LTBI” OR “latent TB”) to search the Web of Science for LTBI-related articles published 1995–2018, then classified the results into three research areas: laboratory sciences, clinical research, and public health. We calculated the proportions of LTBI-related articles in each area to three areas combined, the average rates of LTBI-related to all scientific and TB-related articles, and the average annual percent changes (AAPC) in rates for all countries and for the top 13 countries individually and combined publishing LTBI research.

**Results:**

The proportion of LTBI-related articles increased over time in all research areas, with the highest AAPC in laboratory (38.2%/yr), followed by public health (22.9%/yr) and clinical (15.1%/yr). South Africa (rate ratio [RR] = 8.28, 95% CI 5.68 to 12.08) and India (RR = 2.53, 95% CI 1.74 to 3.69) had higher RRs of overall TB-related articles to all articles, but did not outperform the average of the top 13 countries in the RRs of LTBI-related articles to TB-related articles. Italy (RR = 1.95, 95% CI 1.45 to 2.63), Canada (RR = 1.73, 95% CI 1.28 to 2.34), and Spain (RR = 1.53, 95% CI 1.13 to 2.07) had higher RRs of LTBI-related articles to TB-related articles.

**Conclusions:**

High TB burden countries (TB incidence > 100 per 100,000 population) published more overall TB-related research, whereas low TB burden countries showed greater focus on LTBI. Given the potential benefits, high TB burden countries should consider increasing their emphasis on LTBI-related research.

## Background

Tuberculosis (TB) is a bacterial disease that remains one of the leading causes of mortality worldwide, with an estimated 10 million cases and 1.2 million deaths in 2018 [1]. In 2015, the World Health Organization (WHO) initiated the End TB Strategy, which aims for a 90% reduction in TB incidence and a 95% reduction in TB mortality by the year 2035 [2]. Currently, the global TB incidence is falling at an average rate of about 2% per year, which is not fast enough to meet the milestones set by the End TB Strategy [1]. In 2018, the first-ever United Nations General Assembly high-level meeting on TB endorsed an ambitious and powerful political declaration to accelerate progress toward End TB targets. Notably, the political declaration intensified research and innovation as one of the key strategies to accelerate progress [3]. WHO has developed a ten-year global action framework for TB research to foster high-quality TB research at both national and global levels [4].

One of the targets in the End TB Strategy is 90% preventive treatment coverage, which relates to the detection and treatment of latent TB infection (LTBI) cases prior to their progression to active TB disease [1]. LTBI is defined as a state of persistent immune response to *Mycobacterium tuberculosis* without clinically manifested evidence of active TB. WHO estimates that 23% of the world’s population (1.7 billion people) have LTBI, and a small proportion of these (5–10% of the 1.7 billion people with LTBI) are expected to progress to develop TB disease during their lifetimes [1]. Intensifying efforts to detect and treat LTBI could help reduce the reservoir of potential TB cases and thus contribute to the reduction and eventual elimination of TB incidence.

Global efforts to intensify TB prevention, control, and research activities have resulted in a large number of scientific publications about TB. An increase in research activities has been reported for TB in general [5, 6] and multidrug-resistant TB [7]. These reports used a tool called bibliometric analysis that allows tracking and assessment of research productivity and growth over a period of time. As similar comprehensive assessments have not been done for LTBI research, it is unclear whether the global trend in scientific publications for LTBI has also been increasing. WHO recently published an updated and consolidated guideline for LTBI programmatic management and has also identified research priorities for LTBI [8]. We are interested in whether publication trends specifically for LTBI are on par with WHO’s recommendations, and also whether the research gaps prioritized by WHO are being addressed across countries. This study therefore aimed to investigate the research trends in LTBI at the global level and to analyze gaps in research emphasis in selected countries.

## Methods

### Search terms, data source, and study period

To find LTBI-related research articles, we reviewed previous systematic reviews on LTBI to define our search terms [9, 10]. We then searched the Web of Science™ (Clarivate Analytics) with the search terms (“latent tuberculosis” OR “LTBI” OR “latent TB”) entered under the Topic search field, which searches Title, Abstract, Author Keywords, and Keyword Plus fields [11]. To find overall TB-related research articles, we entered the search terms (“tuberculosis” OR “TB”) under the Title search field, following the methods of a similar study on TB research [5]. Restricting the search to only the Title field helps to minimize false positive search results. We included original scientific articles and reviews (articles, hereinafter) with year of publication during the period 1995–2018. We began the search with the year 1995 for two reasons. First, 1995 is when WHO launched DOTS (or Directly Observed Treatment, Short Course) as the recommended strategy for TB control. Second, prior to 1995, we found no more than three LTBI-related articles published per year [12].

### Research area grouping process

We exported the list of publications resulting from the Web of Science into the InCites™ (Clarivate Analytics) platform for further grouping. Each article was assigned by the Web of Science to one or multiple subject categories [13]. To ensure each subject category could only be assigned to one research area, we then followed the protocol published in a previous paper [13] to group the Web of Science’s subject categories into three research areas [14]: (A) laboratory sciences, which includes fundamental research (or basic science) and vaccines; (B) clinical research, which includes diagnostics and treatments; and (C) public health research, which includes epidemiology, operational research, and public health. All three research areas combined encompass the six research priorities identified by WHO’s international roadmap for TB research [15], namely epidemiology, fundamental research, research and development of new drugs, research and development of new diagnostics, research and development of new vaccines, and operational and public health research. The first step was double-blind classification of subject categories by two researchers (76% agreement). The second step was an independent classification, without knowing assignment results, for disputed cases by a senior researcher and reached 98% agreement. The final step was a meeting among all three researchers to assign the remaining 2% based on consensus. Additional file 1 shows the assignments of subject categories to the three areas: laboratory (*n* = 32), clinical (*n* = 30), and public health (*n* = 47). We excluded subject categories irrelevant to the three research areas from further statistical analysis.

### Statistical analysis

We calculated two rates to compare the number of LTBI-related articles to the number of all articles and to the number of all TB-related articles, respectively. Rate 1 was calculated by first dividing the number of LTBI-related articles (area-specific and all three areas) by the total number of scientific articles (area-specific and all three areas) for each year, and then averaging the results over 24 years and multiplying by 1000. The unit of Rate 1 is ‰ per year. Rate 2 was calculated by replacing the denominator to the number of TB articles and multiplier to 100. Thus, the unit of Rate 2 is % per year.

We calculated the trend in the global proportion of LTBI-related articles out of all published articles (a number available from InCites) and all TB-related articles, respectively, in each research area separately and in the three areas combined, from 1995 through 2018. We hypothesized that a trend may have at most four significant changes [16] and thus applied the joinpoint regression model with autocorrelation errors to evaluate the best number of joinpoints and their locations on a trend [17]. Bayesian information criterion were used to determine the better model with the best number of joinpoints. Additional file 2 shows the test results for number of joinpoints. The model can be expressed as a log-linear regression where the natural logarithm of publication rate (i.e., the number of LTBI-related articles divided by the number of all articles and all TB-related articles) was predicted by a calendar time variable (1 = 1995, 2 = 1996,..., 24 = 2018). When the number of joinpoints and their locations were determined, we estimated two kinds of average annual percent change (AAPC, a weighted average of yearly change [18]) from 1995 to 2018: one is the average percent change per year in the proportion of LTBI-related articles relative to all articles (AAPC 1), and another is the average percent change per year in the proportion of LTBI-related articles relative to all TB-related articles (AAPC 2). Both AAPC 1 and AAPC 2 were derived in each research area and in the three areas combined. The model fitting and AAPC calculations were accomplished using the Joinpoint Regression Program version 4.7.0.0 (National Cancer Institute, United States).

Separately, we selected the top 13 countries publishing LTBI research, which accounted for 80.4% of LTBI-related articles published during the study period, and repeated the above analyses. In addition, we performed the generalized additive mixed model on the data from these 13 countries to examine the heterogeneity in the publication rate of LTBI-related articles relative to all articles and all TB-related articles as well as the rate of TB-related articles relative to all articles, using R studio version 1.0.153 (R Foundation for Statistical Computing, Austria) [19]. Our model can be expressed as the following equation:

$$ In\left({\mu}_{it}\right)=\alpha +{\alpha}_i+f(t)+ offset $$

where *μ*_*it*_ denotes the expected number of LTBI-related articles in country *i* at calendar time *t* following a Poisson distribution; *α* is the fixed intercept, and *α*_*i*_ is the random intercept to account for the initial disparities at the country level; *f*(*t*) denotes a cubic spline to control for temporal autocorrelation [20–22]; and *offset* denotes the natural logarithm of all articles or all TB-related articles in each country per year. The estimated *α*_*i*_ can be transformed by an exponential function to explain the rate ratio (RR) of LBTI-related articles published in one country compared to those published in all 13 countries. The 95% confidence interval of the RR was calculated for each country.

## Results

### Global trends

From 1995 to 2018, a total of 4404 LTBI-related articles were identified in the Web of Science. From these, 4352 articles (98.8%) with information on the year of publication, country of authors, and Web of Science subject categories were imported to InCites and classified into at least one research area.

**Fig. 1:**
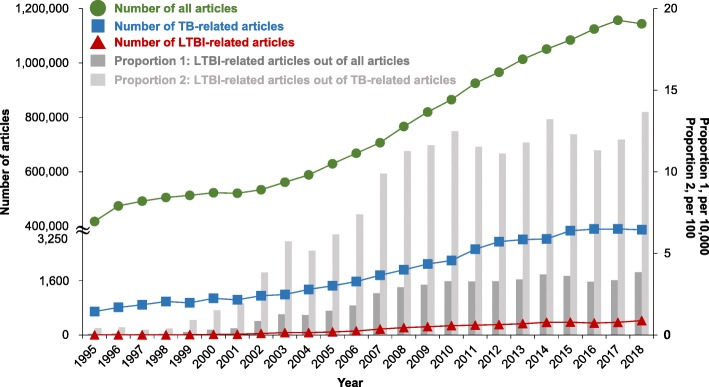
Trend in number and proportion of articles related to latent tuberculosis infection (LTBI), 1995–2018. Articles were defined as articles or reviews belonging to any of the three research areas (see Additional file 1) in InCites™ (Clarivate Analytics)

The number of articles consistently trended upward for all, all TB-related, and specifically LTBI-related research (Fig. 1). The proportion of all articles that were LTBI-related increased from 0.07 per 10,000 in 1995 to 3.84 per 10,000 in 2018. The proportion of TB-related articles that addressed LTBI also increased, from 0.42% in 1995 to 13.66% in 2018. The growth in the proportion of LTBI-related articles (proportion 2 in Fig. 1) was faster from 1995 to 2008 and slowed afterward.

**Fig. 2:**
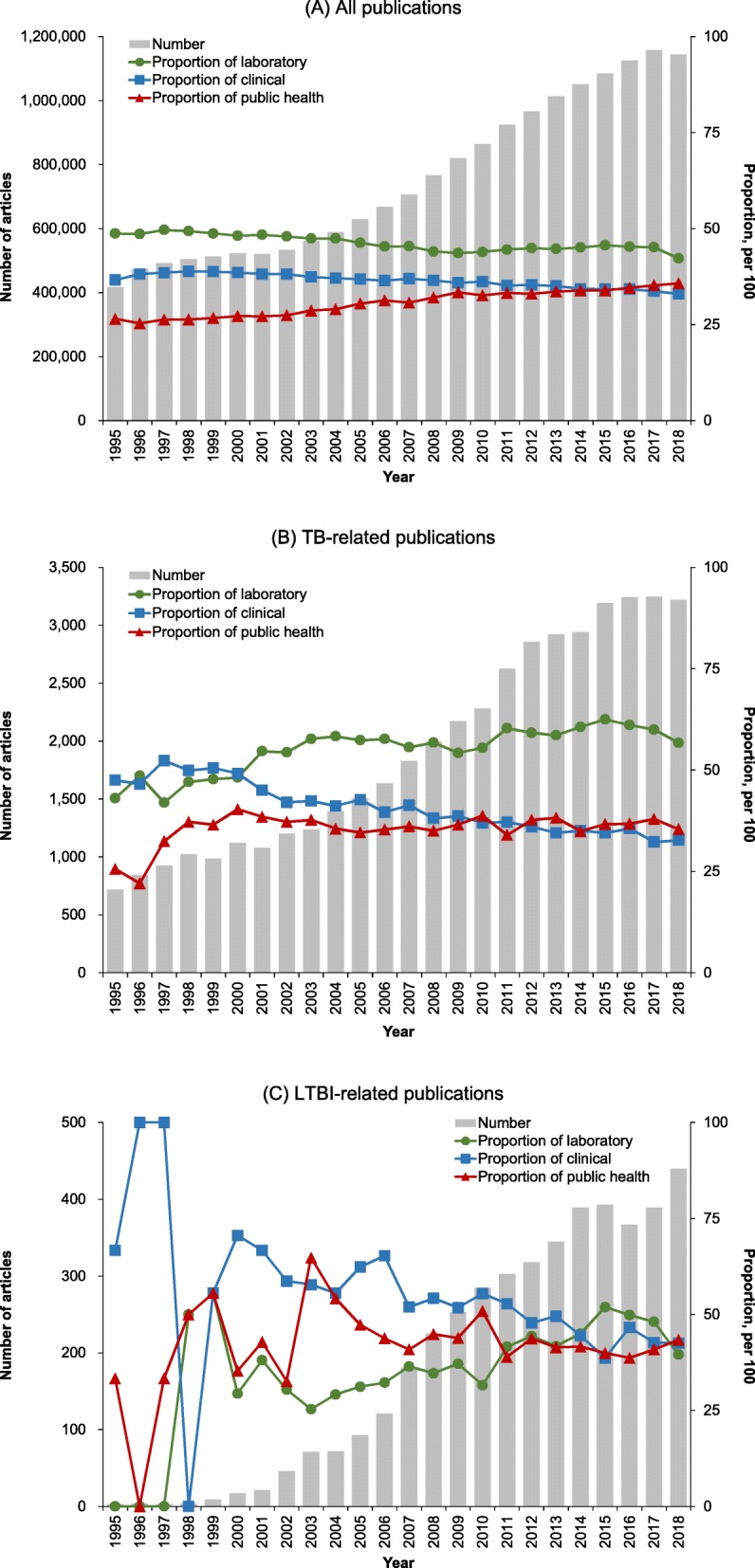
Trends in number and proportion of articles published by research area, 1995–2018. Articles were defined as articles or reviews belonging to any of the three research areas (see Additional file 1 in InCites™ (Clarivate Analytics)

Among all articles published between 1995 and 2018 in all countries in the three research areas, the largest proportion were in the laboratory sciences area, followed by clinical research and public health (Fig. 2a). The proportions of published articles in the laboratory and clinical areas decreased over time, whereas the proportion published in the public health area increased, showing a narrowing gap among the three research areas. For TB-related articles in all countries (Fig. 2b), the laboratory area remained the largest proportion and grew over time, leading to a widening gap between it and the other two areas. For LTBI-related articles in all countries (Fig. 2c), the proportions published in the three areas fluctuated before 2003 due to the small number of articles overall (*N* < 50 for the three research areas combined). Although the proportion in the laboratory area was the smallest until 2011, it grew over time after that and narrowed the gap between laboratory publications and publications in the other two areas. The average proportions of LTBI-related articles during the study period were 41.4% in laboratory, 48.8% in clinical, and 42.8% in public health (Table 1, All countries, Proportion).

**Table 1:** Trends in LTBI-related articles published 1995–2018 overall and in the top 13 countries, by research area

Country	Three areas combined					Laboratory sciences						Clinical research						Public health					
	n ^a^	Rate 1 (‰ per year) ^b^	Rate 2 (% per year) ^c^	AAPC 1 (% per year) ^d^	AAPC 2 (% per year) ^d^	n ^a^	Proportion (%) e	Rate 1 (‰ per year) ^b^	Rate 2 (% per year) ^c^	AAPC 1 (% per year) ^d^	AAPC 2 (% per year) ^d^	n ^a^	Proportion (%) e	Rate 1 (‰ per year) ^b^	Rate 2 (% per year) ^c^	AAPC 1 (% per year) ^d^	AAPC 2 (% per year) ^d^	n ^a^	Proportion (%) e	Rate 1 (‰ per year) ^b^	Rate 2 (% per year) ^c^	AAPC 1 (% per year) ^d^	AAPC 2 (% per year) ^d^
**All countries**	**4352**	**0.20**	**7.4**	**17.8**^f^	**19.2**^f^	**1803**	**41.4**	**0.18**	**5.0**	**38.2**^f^	**32.3**^f^	**2122**	**48.8**	**0.28**	**10.0**	**15.1**^f^	**13.9**^f^	**1863**	**42.8**	**0.26**	**8.8**	**22.9**^f^	**23.0**^f^
**Top 13 countries**	**3499**	**0.21**	**7.7**	**21.8**^f^	**19.1**^f^	**1500**	**42.9**	**0.20**	**5.1**	**18.2**^f^	**14.7**^f^	**1648**	**47.1**	**0.29**	**10.6**	**18.7**^f^	**14.4**^f^	**1542**	**44.1**	**0.29**	**9.4**	**23.4**^f^	**23.3**^f^
United States	1511	0.23	9.8	19.0^f^	17.3^f^	662	43.8	0.24	6.2	16.1^f^	13.1^f^	678	44.9	0.28	14.3	11.8	10.4	756	50.0	0.32	12.2	22.5^f^	21.8^f^
United Kingdom	487	0.26	6.5	16.7	12.7	215	44.1	0.30	5.2	24.2^f^	19.9^f^	207	42.5	0.32	8.5	21.7^f^	18.7^f^	208	42.7	0.27	6.0	10.7	6.8
Italy	303	0.29	13.6	22.4^f^	19.8^f^	125	41.3	0.29	9.5	28.5^f^	26.7^f^	142	46.9	0.31	18.2	16.9^f^	13.5^f^	106	35.0	0.37	14.8	11.8^f^	17.5^g^
Canada	284	0.28	13.4	35.6^f^	29.4^f^	78	27.5	0.19	6.7	15.2^f^	12.2^f^	167	58.8	0.45	22.0	29.4^f^	28.4^f^	146	51.4	0.37	16.7	34.0^f^	11.9
South Africa	254	1.62	5.3	26.5^f^	18.8^f^	129	50.8	2.02	5.4	20.3^f^	13.8^f^	120	47.2	3.52	5.7	19.0	16.3	102	40.2	1.27	4.2	23.6^f^	16.6
Spain	241	0.30	11.2	25.1^f^	27.6^f^	101	41.9	0.27	7.7	27.5^f^	24.0^f^	123	51.0	0.49	15.3	18.8	12.8^f^	116	48.1	0.42	13.9	23.0	24.1^f^
China	240	0.07	2.8	14.7	13.9	151	62.9	0.07	2.6	23.6^f^	19.2^f^	76	31.7	0.09	3.2	13.5^f^	5.0^f^	87	36.3	0.10	4.9	7.3	3.0^fg^
Germany	230	0.14	8.7	24.1^f^	20.9^f^	89	38.7	0.12	4.8	26.7^f^	17.6^f^	99	43.0	0.17	12.6	19.1^f^	15.2^f^	87	37.8	0.19	12.6	23.7^f^	19.6^f^
India	224	0.28	3.0	21.6	10.1^f^	135	60.3	0.26	2.6	29.6^f^	12.0^f^	90	40.2	0.50	3.3	14.0	14.1	68	30.4	0.37	3.8	13.7	9.1
Brazil	190	0.32	6.4	24.7^f^	18.8^f^	72	37.9	0.25	4.7	17.1^f^	13.6^f^	91	47.9	0.42	9.2	15.0	17.3^f^	97	51.1	0.50	6.2	24.1^f^	20.1^f^
France	185	0.17	5.1	35.9^f^	23.1	67	36.2	0.13	3.0	20.1	14.8	99	53.5	0.27	8.2	31.5^f^	33.2^f^	73	39.5	0.21	5.4	25.0^f^	25.2^f^
Netherlands	183	0.28	7.6	19.8^f^	16.3^f^	80	43.7	0.34	6.2	21.3^f^	16.3^f^	89	48.6	0.33	9.7	15.5^f^	10.9	78	42.6	0.36	6.8	17.4	13.9
South Korea	173	0.23	8.1	20.1^f^	18.2^f^	60	34.7	0.15	5.6	15.1^f^	13.3^f^	106	61.3	0.37	10.1	10.4^f^	12.5^f^	61	35.3	0.38	11.2	7.7	11.3^g^

The orders of the top 13 countries are listed based on the total number of LTBI-related articles published 1995–2018

^a^ n = Number of LTBI-related articles published 1995–2018, defined as articles or reviews belonging to any of three research areas (see Additional file 1) in InCites™ (Clarivate Analytics). Because each article may be assigned to more than one research area and country in InCites, duplicates were excluded when the three areas were combined

^b^ Rate 1 (unit: ‰ per year) was calculated by first dividing the number of LTBI-related articles (area-specific and all three areas) by the total number of scientific articles (area-specific and all three areas) for each year, and then averaging the results over 24 years and multiplying by 1000 (thus the unit is ‰ per year, which is a rate rather than a proportion)

^**c**^ Rate 2 (unit: % per year) was calculated by first dividing the number of LTBI-related articles (area-specific and all three areas) by the number of TB articles (area-specific and all three areas) for each year, and then averaging the results over 24 years and multiplying by 100 (thus the unit is % per year, which is a rate rather than a proportion)

^d^ AAPC = Average annual percent change (unit: % per year). AAPC 1 represents the average percent change per year in the proportion of LTBI-related articles relative to all articles. AAPC 2 represents the average percent change per year in the proportion of LTBI-related articles relative to all TB-related articles

^e^ Proportion (unit: %) was calculated by dividing the number of area-specific LTBI-related articles by the number of LTBI-related articles across all three areas, over 24 years. Because the sum of the number of articles in each of the three research areas exceeded the number of articles across all three areas (excluding duplicates), the sum of the proportions of the three research areas may exceed 100%

^f^*p*-value less than 0.05

^g^ Italy and China starting from 1996. South Korea starting from 1997

When we compared the number of LTBI-related articles to the number of all articles, 1995–2018, the average rates were highest in the clinical research area (0.28‰/yr), followed by public health (0.26‰/yr) and laboratory sciences (0.18‰/yr) areas (Table 1, Rate 1). In growth over time, the AAPC showed a significant increase in all three areas, with the highest AAPC in laboratory (38.2%/yr), followed by public health (22.9%/yr) and clinical (15.1%/yr) (Table 1, AAPC 1). Similar patterns were observed when we compared the number of LTBI-related articles to the number of all TB-related articles (Table 1, Rate 2 and AAPC 2).

### Top 13 countries

For the average number of LTBI-related articles to all articles published in the top 13 countries, we found similar patterns in proportions (clinical > public health > laboratory), rates (clinical > public health > laboratory), but different patterns for AAPC 1 (public health > clinical > laboratory) and AAPC 2 (public health > laboratory > clinical) (Table 1).

We found diverse patterns for the individual countries, however. When we compared the proportions of LTBI publications by country, the clinical area was dominant in seven countries (led by South Korea’s 61.3%), laboratory dominant in four countries (led by China’s 62.9%), and public health dominant in two countries (the United States and Brazil). We identified five countries with one research area that had a proportion either 10% lower or 10% higher than those of the other two research areas: Canada, with a lower proportion of laboratory publications (27.5%); China and India, with higher proportions of laboratory publications (62.9 and 60.3%); and France and South Korea, with higher proportions of clinical publications (53.5 and 61.3%).

Among the 13 countries, seven had the highest rates of LTBI-related articles to all articles in the public health area, while the other six had the highest rates in the clinical area (Table 1, Rate 1). For the rate of LTBI-related articles to all TB-related articles (Table 1, Rate 2), only three countries had the highest rates of LTBI-related articles in public health, whereas the other ten countries had the highest rates in clinical (led by Canada at 22.0%/yr).

The AAPC of LTBI-related articles relative to all articles among the top 13 countries (Table 1, AAPC 1) showed significant increases in 12 countries in laboratory, eight countries in clinical, and seven countries in public health. As for the AAPC of LTBI-related relative to TB-related articles (Table 1, AAPC 2), significant increases were observed in 12 countries in laboratory, nine countries in clinical, and six countries in public health.

**Fig. 3:**
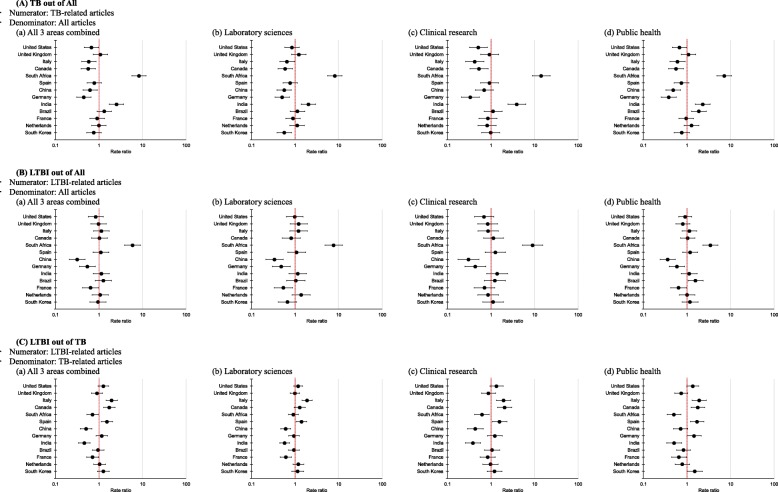
Rate ratios of TB- and LTBI-related articles in each research area by country, compared to the average across countries. The orders of the 13 countries are listed based on the total number of LTBI-related articles published 1995–2018 (in line with Table 1). The red dotted line represents the average across the 13 countries with the highest publication numbers. We estimated the rate ratios by using a generalized additive mixed model with a Poisson distribution and comparing the rate of LTBI-related articles in each research area in each country, and across all three research areas combined, to the average rate across the 13 countries combined, i.e., rate ratio = 1, after adjusting for temporal variation

Figure 3A shows the RR in TB-related articles out of all articles for each of the individual 13 countries compared to the average rate across the 13 countries, for each research area (Fig. 3Ab, 3Ac, 3Ad) and in the three areas combined (Fig. 3Aa), after controlling for temporal variation. Countries with significantly higher RRs included South Africa (in the three research areas combined with RR = 8.28, 95% CI 5.68 to 12.08 and in the individual areas), India (in all three areas combined with RR = 2.53, 95% CI 1.74 to 3.69 and the individual areas), and Brazil (in public health). The lowest RR was observed in Germany, across all research areas.

When the LTBI-related articles were analyzed (Fig. 3B), South Africa had the highest RRs and China had the lowest RRs across all research areas. Figure 3C shows the RR in LTBI-related articles out of all TB-related articles, and here we observed different patterns: Countries with significantly higher RRs included Italy (in the three areas combined with RR = 1.95, 95% CI 1.45 to 2.63 and the individual areas), Spain (in the three areas combined with RR = 1.53, 95% CI 1.13 to 2.07 and the individual areas), Canada (in the three areas combined, clinical, and public health), and South Korea (in public health). The lowest RRs were observed in India (in the three areas combined, laboratory, and clinical) and South Africa (in public health).

## Discussion

We conducted a bibliometric analysis on LTBI-related articles, analyzing publication trends (both globally and among the top 13 countries) of LTBI-related articles relative to all scientific and all TB-related publications from 1995 to 2018. Globally, we found that the average proportions of LTBI-related publications against both all scientific and all TB-related publications were similar in all three research areas (laboratory, clinical, and public health), ranging between 41 and 49%. LTBI-related publications in all three areas significantly increased over time. This could be partly due to the challenges of LTBI case detection and ascertainment [23]. People with LTBI tend to be asymptomatic, with high rates of infection occurring among specific risk groups, particularly household contacts of TB cases [8]. Contact investigation at the community or population level is thus necessary to identify candidates for LTBI testing and treatment [8]. Identifying and initiating treatment among groups of people at high risk of developing TB disease is one of the priorities for TB elimination [3].

All the top 13 countries that we have identified as being involved in most LTBI-related research have also contributed funding to TB research and development (R&D), either as an individual country or as part of an association, such as the European Union or BRICS (Brazil, Russia, India, China, and South Africa) [24]. Our findings are also in line with a previous bibliometric analysis of overall TB research that found the same countries to be also the top publishing countries for overall TB research [5]. In 2017, the largest funder for TB R&D was the United States, followed by the European Union and the United Kingdom [24]. The list of top funders also includes four BRICS countries (i.e., South Africa, China, India, and Brazil). BRICS accounted for 53% of the global funding for TB and 47% of the world’s notified TB cases in 2018 [1]. South Africa, China, India, and Brazil are also part of the top 30 high TB burden countries (i.e., those with TB incidence of > 100 per 100,000 population) as defined by WHO [25].

Even though BRICS countries are in the top 13 countries for LTBI-related research, further analysis found that they in fact placed more emphasis on TB research than specifically on LTBI research. In particular, South Africa, India, and Brazil had significantly higher RRs for TB-related publications relative to all scientific publications. One possible explanation lies in the TB burden of each country: All three countries have high TB incidence rates (520, 199, and 45 per 100,000 population in South Africa, India, and Brazil, respectively in 2018) and are among the top 30 high TB burden countries [25]. All three countries also have a high burden of HIV, and the presence of this infection is known to be a predisposing and precipitating factor for the development of TB [23]. TB is a major occupational disease among mineworkers in South Africa, whose TB incidence rates are 3 to 7 times higher than that of the country’s general population [26]. Recognizing the importance of TB, these countries have invested significantly in TB R&D. India has publicly recognized the financial implications of the TB burden in the country, and its government has pledged to fund TB research activities [27]. South Africa invests more than 0.1% of the country’s gross domestic product to TB R&D (called the 0.1% fair share target) [24]. This could explain why we found high RRs of both TB- and LTBI-related research, relative to all scientific publications, in South Africa.

Also, we found that South Africa and India had significantly low RRs for LTBI-related research publications relative to all TB-related publications. In addition to being high TB burden countries as mentioned earlier, both countries are also among the top TB/HIV (incidence rate of 306 and 6.8 per 100,000 population, for South Africa and India respectively) and multidrug-resistant TB (incidence rate of 19 and 9.6 per 100,000 population, for South Africa and India respectively) burden countries [1]. Hence, this finding could be due to them prioritizing TB disease and not LTBI. Yet one reason for the slow progress against TB is the presence of a huge and persistent reservoir of LTBI. Despite the fact that most high and low TB burden countries already have a national policy addressing LTBI management in specific risk groups, most TB control programs in low- and middle-income countries have focused almost exclusively on detection and treatment of active TB cases [3]. Taken together, our findings suggest that high TB burden countries tend to prioritize TB-related research, which is unsurprising given the challenges of TB treatment and control in such countries. These countries also need to tackle issues of high HIV incidence, which is a precipitating factor in developing TB disease.

When using all TB-related publications as the denominator for RR analysis, we observed that Canada, Italy, and Spain had significantly higher RRs of LTBI-related publications for the three research areas combined. All three countries have been identified as low TB burden countries (i.e., those with TB incidence of < 10 per 100,000 population) based on recent surveillance reports [28, 29]. As proposed in WHO’s framework towards TB elimination for low-incidence countries [30], preventing the progression of LTBI to TB disease (through detection and early treatment of LTBI cases) will play a crucial role in eliminating TB in these countries. This could explain these countries’ emphasis on LTBI research. In particular, Italy has highlighted the prioritization of LTBI testing and treatment in high-risk groups as one of its eight main interventions to eliminate TB [31].

From the same analysis, we observed high RRs of LTBI compared to TB publications for South Korea in the public health area. South Korea is the only Organisation for Economic Co-operation and Development country with high TB incidence, and since 2013 it has been implementing a five-year TB control plan that emphasizes extensive contact investigation and LTBI management [32]. This could explain the increase in public health publications on LTBI in South Korea.

Although we observed an overall increasing trend of LTBI-related publications for the public health area, comparing the Rate 2 results across the three research areas in each of the top 13 countries tells us another story. That is, only three countries (China, India, and South Korea) have highest rates (4.9, 3.8, and 11.2% per year, respectively) for public health-related research among the three research areas analyzed. As mentioned earlier, community studies are important to determine the burden of LTBI to allow the implementation of locally tailored interventions. Taken together, our findings suggest that more studies focusing on LTBI are needed, particularly on public health-related research, not only in low TB burden countries but also in high TB burden countries with rapidly expanding economies. Indeed, determining the burden of LTBI in various geographical setting and identifying population groups at-risk of progression to TB disease are the two research public health-related priorities highlighted by WHO [3]. Modeling studies have shown that implementing a combination of interventions (i.e., to prevent TB infections in addition to detecting and treating TB patients) results in major reductions in TB burden [33, 34] and thus helps to reach the targets set by the End TB Strategy.

This study has several limitations. First are the methodology limitations mentioned in a previous bibliometric analysis [13], including potential misclassifications when assigning articles into research areas or countries as well as underappreciation of countries that have only recently begun TB research and with few accumulated publications. We could reduce misclassification bias by assigning each article to multiple subject categories and multiple countries, but bear the side effect that the sum of the number of articles in the three research areas and multiple countries would exceed the sum of the three-areas-combined and the all-countries-combined [13]. We have limited information for countries just begun TB research in recent years. Similarly, countries published research findings in non-English journals or new journals without impact factors were not captured in our analysis. We suggest a separate analysis after a certain period of follow-up to consider these potential changes in the future. Next, for this study we classified articles into three broad research areas instead of the six specific research priorities identified by WHO’s international roadmap for TB research [15]. Our classification is not fully representative of each priority in the roadmap, but is still in line: the laboratory sciences area covers the priorities of fundamental research and vaccines, the clinical research area covers diagnosis and treatment, and the public health area covers epidemiology and operation and public health research. Hence, the broadly categorized trends observed in our study could still reflect the research trends proposed by WHO. Using fewer categories, meanwhile, increased the number of LTBI papers in each area and allowed us to perform statistical analyses. Finally, we extracted data on the 13 countries that had the most publications regarding LTBI. However, TB burden generally affects developing countries, where resources for research could be scarce, and this might limit the generalizability of our interpretation to these countries.

## Conclusions

Globally, there has been positive progress towards more LTBI-related research, with the number of publications growing annually from 1995 to 2018. Discrepancies across countries exist in the emphasis on either TB or LTBI research. High TB burden countries have been more involved in overall TB-related research, whereas low TB burden countries have focused more on LTBI-related research. Given the potential benefits of LTBI research to reducing TB incidence, our findings suggest that high TB burden countries should place more emphasis on research related to LTBI.

## Supplementary information

**Additional file 1.**

**Additional file 2.**

## Data Availability

The datasets generated and analyzed during the current study are available in the Web of Science (https://apps.webofknowledge.com) and InCites (https://incites.clarivate.com). The datasets used and analyzed during the current study are also available from the corresponding author on reasonable request.

## Abbreviations

AAPC: Average annual percent changes
DOTS: Directly Observed Treatment, Short Course
LTBI: Latent tuberculosis infection
RR: Rate ratio
TB: Tuberculosis
WHO: World Health Organization
