# Supplementary material for: Global trends and gaps in research related to latent tuberculosis infection
Source: BMC Public Health. 2020 Mar 18;20:352. doi: 10.1186/s12889-020-8419-0 (PMC7079542; doi:10.1186/s12889-020-8419-0)
Supplement: Supplementary file 1 — Additional file 1. [file 12889_2020_8419_MOESM1_ESM.pdf]

Additional file 1

There are 254 subject categories in the Web of Science. Table S1 shows how the subject categories were assigned to each of the three research areas of interest in this study: laboratory sciences (n = 32), clinical research (n = 30), and public health (n = 47).

Table S1. Assignment of subject categories in the Web of Science™ to three research areas

Research area	***Subject category***
(1) Laboratory sciences	Allergy
	Biochemical Research Methods
	Biochemistry & Molecular Biology
	Biology
	Biophysics
	Biotechnology & Applied Microbiology
	Cell & Tissue Engineering
	Cell Biology
	Chemistry, Analytical
	Chemistry, Applied
	Chemistry, Inorganic & Nuclear
	Chemistry, Medicinal
	Chemistry, Multidisciplinary
	Chemistry, Organic
	Chemistry, Physical
	Engineering, Biomedical
	Evolutionary Biology
	Genetics & Heredity
	Hematology
	Immunology
	Mathematical & Computational Biology
	Medical Informatics
	Medical Laboratory Technology
	Medicine, Research & Experimental
	Microbiology
	Microscopy
	Multidisciplinary Sciences
	Parasitology
	Pathology
	Physiology
	Veterinary Sciences
	Virology
(2) Clinical research	Cardiac & Cardiovascular Systems
	Clinical Neurology
	Critical Care Medicine
	Dentistry, Oral Surgery & Medicine
	Dermatology
	Emergency Medicine
	Endocrinology & Metabolism
	Gastroenterology & Hepatology
	Geriatrics & Gerontology
	Imaging Science & Photographic Technology
	Integrative & Complementary Medicine
	Medicine, General & Internal
	Neurosciences
	Nutrition & Dietetics
	Obstetrics & Gynecology
	Oncology
	Ophthalmology
	Orthopedics
	Otorhinolaryngology
	Pediatrics
	Peripheral Vascular Disease

	Pharmacology & Pharmacy
	Primary Health Care
	Radiology, Nuclear Medicine & Medical Imaging
	Respiratory System
	Rheumatology
	Surgery
	Toxicology
	Transplantation
	Urology & Nephrology
(3) Public health	Anthropology
	Area Studies
	Behavioral Sciences
	Communication
	Computer Science, Artificial Intelligence
	Computer Science, Information Systems
	Computer Science, Interdisciplinary Applications
	Cultural Studies
	Demography
	Ecology
	Economics
	Education & Educational Research
	Education, Scientific Disciplines
	Education, Special
	Engineering, Environmental
	Engineering, Geological
	Environmental Sciences
	Environmental Studies
	Ethics
	Ethnic Studies
	Family Studies
	Geography
	Gerontology
	Health Care Sciences & Services
	Health Policy & Services
	Infectious Diseases
	Law
	Mathematics
	Mathematics, Applied
	Mathematics, Interdisciplinary Applications
	Medical Ethics
	Meteorology & Atmospheric Sciences
	Nursing
	Psychiatry
	Psychology
	Psychology, Social
	Public Administration
	Public, Environmental & Occupational Health
	Social Issues
	Social Sciences, Biomedical
	Social Sciences, Interdisciplinary
	Social Work
	Sociology
	Statistics & Probability
	Substance Abuse
	Tropical Medicine
	Urban Studies
