# Supplementary material for: Global trends and gaps in research related to latent tuberculosis infection
Source: BMC Public Health. 2020 Mar 18;20:352. doi: 10.1186/s12889-020-8419-0 (PMC7079542; doi:10.1186/s12889-020-8419-0)
Supplement: Supplementary file 2 — Additional file 2. [file 12889_2020_8419_MOESM2_ESM.pdf]

Additional file 2

Table S2.1. The Bayesian information criterion (BIC) in the joinpoint regression. The model with the smallest BIC was selected for AAPC 1 estimation in each research discipline.

Country	*Research area*	*Number of joinpoint(s)*	*BIC*	*Selected joinpoint(s)*
(1) All countries	Three areas combined	0	3.205	
		1	1.210	
		2	0.710	
		**3**	**0.703**	**3**
		4	0.820	
	Laboratory sciences	0	1.936	
		1	0.307	
		2	-0.171	
		**3**	**-0.178**	**3**
		4	0.042	
	Clinical research	0	3.291	
		1	1.044	
		2	0.570	
		3	0.546	
		**4**	**0.535**	**4**
	Public health	0	2.964	
		1	1.836	
		**2**	**0.959**	**2**
		3	1.011	
		4	1.107	
(2) Top 13 countries	Three areas combined	0	2.868	
		1	1.288	
		**2**	**0.609**	**2**
		3	0.714	
		4	0.874	
	Laboratory sciences	0	1.638	
		1	0.422	
		**2**	**-0.010**	**2**
		3	0.002	
		4	0.233	
	Clinical research	0	2.900	
		1	1.064	
		**2**	**0.524**	**2**
		3	0.604	
		4	0.602	
	Public health	0	3.069	
		1	1.788	
		**2**	**1.164**	**2**
		3	1.255	
		4	1.300	
(3) United States	Three areas combined	0	2.243	
		1	0.555	
		**2**	**0.513**	**2**
		3	0.724	
		4	0.935	
	Laboratory sciences	0	0.775	
		1	0.215	
		**2**	**0.201**	**2**
		3	0.298	
		4	0.425	
	Clinical research	0	1.993	
		1	0.339	
		2	0.274	
		**3**	**0.261**	**3**
		4	0.412	
	Public health	0	2.281	

		**1**	**0.754**	**1**
		2	0.963	
		3	1.110	
		4	1.207	
(4) United Kingdom	Three areas combined	0	2.079	
		1	0.513	
		2	0.465	
		**3**	**0.384**	**3**
		4	0.435	
	Laboratory sciences	0	1.374	
		**1**	**0.580**	**1**
		2	0.759	
		3	0.794	
		4	0.993	
	Clinical research	0	1.594	
		1	-0.032	
		**2**	**-0.249**	**2**
		3	-0.156	
		4	0.032	
	Public health	0	1.118	
		1	0.806	
		2	0.563	
		3	0.061	
		**4**	**0.055**	**4**
(5) Italy	Three areas combined	0	1.254	
		**1**	**0.263**	**1**
		2	0.444	
		3	0.565	
		4	0.778	
	Laboratory sciences	0	0.415	
		**1**	**-0.649**	**1**
		2	-0.541	
		3	-0.423	
		4	-0.205	
	Clinical research	0	1.077	
		**1**	**0.536**	**1**
		2	0.697	
		3	0.719	
		4	0.824	
	Public health	0	0.790	
		**1**	**0.304**	**1**
		2	0.397	
		3	0.589	
		4	0.550	
(6) Canada	Three areas combined	0	1.422	
		1	0.836	
		**2**	**0.644**	**2**
		3	0.875	
		4	1.053	
	Laboratory sciences	0	-0.335	
		**1**	**-1.139**	**1**
		2	-1.029	
		3	-0.858	
		4	-0.661	
	Clinical research	0	1.335	
		1	0.642	
		**2**	**0.525**	**2**
		3	0.706	
		4	0.807	
	Public health	0	0.896	
		1	0.482	
		2	0.501	
		**3**	**0.317**	**3**

		4	0.346	
(7) South Africa	Three areas combined	0	0.660	
		**1**	**-0.081**	**1**
		2	0.103	
		3	0.346	
		4	0.582	
	Laboratory sciences	0	0.287	
		**1**	**-0.185**	**1**
		2	-0.003	
		3	0.080	
		4	0.279	
	Clinical research	0	0.733	
		1	-0.342	
		2	-0.399	
		**3**	**-0.830**	**3**
		4	-0.751	
	Public health	0	0.383	
		**1**	**-0.191**	**1**
		2	0.009	
		3	0.179	
		4	0.359	
(8) Spain	Three areas combined	0	1.973	
		1	1.289	
		2	0.977	
		3	0.855	
		**4**	**0.812**	**4**
	Laboratory sciences	0	0.575	
		1	0.499	
		2	0.432	
		3	0.419	
		**4**	**0.260**	**4**
	Clinical research	0	0.651	
		**1**	**0.620**	**1**
		2	0.693	
		3	0.672	
		4	0.755	
	Public health	0	0.761	
		1	0.296	
		2	0.229	
		3	0.113	
		**4**	**-0.066**	**4**
(9) China	Three areas combined	0	0.335	
		1	-0.718	
		2	-0.925	
		**3**	**-0.999**	**3**
		4	-0.753	
	Laboratory sciences	0	0.250	
		**1**	**-0.136**	**1**
		2	-0.115	
		3	0.030	
		4	0.257	
	Clinical research	0	0.246	
		**1**	**0.185**	**1**
		2	0.226	
		3	0.344	
		4	0.572	
	Public health	0	-0.473	
		1	-1.236	
		2	-1.229	
		**3**	**-1.262**	**3**
		4	-1.007	
(10) Germany	Three areas combined	0	2.085	
		**1**	**0.588**	**1**

		2	0.715	
		3	0.729	
		4	0.955	
	Laboratory sciences	0	1.286	
		1	0.953	
		2	0.959	
		**3**	**0.563**	**3**
		4	0.790	
	Clinical research	0	1.105	
		**1**	**-0.180**	**1**
		2	-0.036	
		3	0.092	
		4	0.333	
	Public health	0	0.378	
		**1**	**0.071**	**1**
		2	0.159	
		3	0.402	
		4	0.554	
(11) India	Three areas combined	0	0.069	
		1	-0.067	
		2	0.019	
		3	-0.174	
		**4**	**-0.394**	**4**
	Laboratory sciences	0	-0.010	
		1	0.031	
		2	0.175	
		**3**	**-0.099**	**3**
		4	-0.015	
	Clinical research	0	1.101	
		1	1.145	
		2	1.240	
		3	0.837	
		**4**	**0.741**	**4**
	Public health	0	0.388	
		1	-0.423	
		2	-0.648	
		**3**	**-0.809**	**3**
		4	-0.600	
(12) Brazil	Three areas combined	0	1.053	
		**1**	**0.119**	**1**
		2	0.217	
		3	0.329	
		4	0.578	
	Laboratory sciences	0	0.688	
		1	0.301	
		2	0.255	
		**3**	**-0.318**	**3**
		4	-0.110	
	Clinical research	0	0.516	
		1	0.064	
		2	-0.216	
		**3**	**-0.260**	**3**
		4	-0.099	
	Public health	0	0.008	
		1	-0.267	
		2	-0.525	
		**3**	**-0.757**	**3**
		4	-0.732	
(13) France	Three areas combined	0	1.639	
		1	0.440	
		2	0.171	
		**3**	**-0.144**	**3**
		4	-0.085	

	Laboratory sciences	0	0.682	
		1	-0.202	
		2	-0.372	
		**3**	**-0.837**	**3**
		4	-0.689	
	Clinical research	0	1.286	
		1	1.048	
		2	0.347	
		**3**	**-0.396**	**3**
		4	-0.228	
	Public health	0	0.362	
		**1**	**-0.219**	**1**
		2	-0.056	
		3	0.071	
		4	0.277	
(14) Netherlands	Three areas combined	0	1.615	
		**1**	**0.857**	**1**
		2	0.953	
		3	0.927	
		4	1.075	
	Laboratory sciences	0	0.321	
		**1**	**-0.174**	**1**
		2	-0.022	
		3	0.141	
		4	0.356	
	Clinical research	0	0.434	
		**1**	**-0.147**	**1**
		2	-0.019	
		3	0.122	
		4	0.258	
	Public health	0	1.422	
		1	0.781	
		2	0.680	
		**3**	**0.498**	**3**
		4	0.645	
(15) South Korea	Three areas combined	0	1.176	
		**1**	**0.435**	**1**
		2	0.539	
		3	0.611	
		4	0.783	
	Laboratory sciences	0	0.893	
		1	-0.478	
		**2**	**-0.505**	**2**
		3	-0.339	
		4	-0.399	
	Clinical research	**0**	**0.225**	**0**
		1	0.321	
		2	0.457	
		3	0.696	
		4	0.806	
	Public health	0	0.772	
		1	-0.253	
		2	-0.298	
		**3**	**-0.505**	**3**
		4	-0.389	

Table S2.2. The Bayesian information criterion (BIC) in the joinpoint regression. The model with the smallest BIC was selected for AAPC 2 estimation in each research discipline.

Country	*Research area*	*Number of joinpoint(s)*	*BIC*	*Selected joinpoint(s)*
(1) All countries	Three areas combined	0	2.976	
		1	1.201	
		**2**	**0.798**	**2**
		3	0.832	
		4	0.991	
	Laboratory sciences	0	1.486	
		1	-0.219	
		2	-0.266	
		**3**	**-0.277**	**3**
		4	-0.136	
	Clinical research	0	3.189	
		1	1.307	
		2	1.065	
		3	0.941	
		**4**	**0.737**	**4**
	Public health	0	2.953	
		1	1.822	
		2	1.332	
		**3**	**1.293**	**3**
		4	1.457	
(2) Top 13 countries	Three areas combined	0	2.793	
		1	1.396	
		**2**	**0.811**	**2**
		3	0.963	
		4	1.100	
	Laboratory sciences	0	1.177	
		1	-0.060	
		**2**	**-0.160**	**2**
		3	-0.132	
		4	-0.072	
	Clinical research	0	2.875	
		1	1.378	
		2	0.916	
		3	0.756	
		**4**	**0.643**	**4**
	Public health	0	3.117	
		1	1.877	
		2	1.666	
		**3**	**1.616**	**3**
		4	1.809	
(3) United States	Three areas combined	0	2.262	
		**1**	**0.586**	**1**
		2	0.689	
		3	0.931	
		4	1.117	
	Laboratory sciences	0	0.607	
		**1**	**0.083**	**1**
		2	0.237	
		3	0.236	
		4	0.343	
	Clinical research	0	2.282	
		1	0.691	
		**2**	**0.671**	**2**
		3	0.767	
		4	0.832	
	Public health	0	2.661	
		**1**	**1.004**	**1**
		2	1.197	
		3	1.289	
		4	1.514	

(4) United Kingdom	Three areas combined	0	1.977	
		1	0.426	
		2	0.368	
		**3**	**0.210**	**3**
		4	0.278	
	Laboratory sciences	0	1.352	
		**1**	**0.449**	**1**
		2	0.592	
		3	0.551	
		4	0.651	
	Clinical research	0	1.595	
		**1**	**0.237**	**1**
		2	0.294	
		3	0.343	
		4	0.587	
	Public health	0	1.169	
		1	0.932	
		2	0.649	
		**3**	**0.015**	**3**
		4	0.153	
(5) Italy	Three areas combined	0	1.085	
		**1**	**-0.328**	**1**
		2	-0.093	
		3	0.046	
		4	0.250	
	Laboratory sciences	0	0.355	
		1	-0.867	
		**2**	**-0.895**	**2**
		3	-0.846	
		4	-0.713	
	Clinical research	0	1.204	
		**1**	**0.374**	**1**
		2	0.548	
		3	0.573	
		4	0.651	
	Public health	0	0.991	
		1	0.256	
		2	0.225	
		3	0.274	
		**4**	**0.221**	**4**
(6) Canada	Three areas combined	0	1.362	
		1	0.930	
		**2**	**0.911**	**2**
		3	0.931	
		4	1.110	
	Laboratory sciences	0	-0.282	
		**1**	**-0.684**	**1**
		2	-0.501	
		3	-0.302	
		4	-0.112	
	Clinical research	0	1.066	
		1	0.478	
		2	0.541	
		**3**	**0.231**	**3**
		4	0.259	
	Public health	0	1.204	
		1	1.039	
		2	0.799	
		3	0.665	
		**4**	**0.649**	**4**
(7) South Africa	Three areas combined	0	0.303	
		**1**	**-0.490**	**1**
		2	-0.275	

		3	-0.088	
		4	0.171	
	Laboratory sciences	0	0.125	
		1	-0.252	
		**2**	**-0.301**	**2**
		3	-0.145	
		4	-0.016	
	Clinical research	0	0.222	
		1	-0.665	
		**2**	**-0.677**	**2**
		3	-0.553	
		4	-0.326	
	Public health	0	-0.010	
		**1**	**-0.469**	**1**
		2	-0.339	
		3	-0.147	
		4	0.081	
(8) Spain	Three areas combined	0	1.913	
		1	1.112	
		2	0.843	
		3	0.673	
		**4**	**0.445**	**4**
	Laboratory sciences	0	0.478	
		1	0.351	
		2	0.193	
		3	0.122	
		**4**	**-0.007**	**4**
	Clinical research	0	0.851	
		**1**	**0.529**	**1**
		2	0.568	
		3	0.676	
		4	0.707	
	Public health	0	0.846	
		1	-0.252	
		2	-0.283	
		3	-0.325	
		**4**	**-0.513**	**4**
(9) China	Three areas combined	0	-0.210	
		1	-0.821	
		**2**	**-0.842**	**2**
		3	-0.681	
		4	-0.654	
	Laboratory sciences	0	-0.098	
		**1**	**-0.287**	**1**
		2	-0.280	
		3	-0.097	
		4	-0.077	
	Clinical research	**0**	**-0.080**	**0**
		1	0.117	
		2	0.038	
		3	0.236	
		4	0.486	
	Public health	**0**	**-1.100**	**0**
		1	-1.013	
		2	-0.950	
		3	-0.721	
		4	-0.578	
(10) Germany	Three areas combined	0	2.044	
		**1**	**0.710**	**1**
		2	0.855	
		3	0.929	
		4	1.047	
	Laboratory sciences	0	0.978	

		1	0.724	
		2	0.761	
		**3**	**0.481**	**3**
		4	0.734	
	Clinical research	0	1.366	
		**1**	**0.084**	**1**
		2	0.212	
		3	0.440	
		4	0.573	
	Public health	0	0.492	
		1	0.139	
		**2**	**0.112**	**2**
		3	0.326	
		4	0.444	
(11) India	Three areas combined	**0**	**-0.298**	**0**
		1	-0.290	
		2	-0.229	
		3	-0.159	
		4	-0.240	
	Laboratory sciences	**0**	**-0.522**	**0**
		1	-0.366	
		2	-0.173	
		3	-0.256	
		4	-0.056	
	Clinical research	0	0.727	
		1	0.706	
		2	0.753	
		3	0.556	
		**4**	**0.321**	**4**
	Public health	0	0.180	
		1	-0.048	
		2	-0.226	
		**3**	**-0.532**	**3**
		4	-0.324	
(12) Brazil	Three areas combined	0	0.770	
		**1**	**0.303**	**1**
		2	0.524	
		3	0.524	
		4	0.778	
	Laboratory sciences	0	0.639	
		1	0.318	
		2	0.114	
		**3**	**-0.385**	**3**
		4	-0.202	
	Clinical research	0	-0.245	
		**1**	**-0.778**	**1**
		2	-0.682	
		3	-0.481	
		4	-0.296	
	Public health	0	-0.269	
		1	-0.171	
		2	-0.378	
		**3**	**-0.623**	**3**
		4	-0.489	
(13) France	Three areas combined	0	1.428	
		1	0.360	
		2	-0.031	
		3	-0.445	
		**4**	**-0.465**	**4**
	Laboratory sciences	0	0.355	
		1	-0.430	
		2	-0.554	
		**3**	**-0.896**	**3**

		4	-0.640	
	Clinical research	0	1.762	
		1	1.537	
		2	0.738	
		**3**	**0.067**	**3**
		4	0.171	
	Public health	0	0.285	
		**1**	**-0.086**	**1**
		2	0.077	
		3	0.306	
		4	0.450	
(14) Netherlands	Three areas combined	0	1.447	
		1	0.925	
		2	1.073	
		**3**	**0.675**	**3**
		4	0.785	
	Laboratory sciences	0	0.225	
		**1**	**-0.273**	**1**
		2	-0.172	
		3	-0.175	
		4	-0.015	
	Clinical research	0	0.884	
		1	0.309	
		2	0.343	
		**3**	**0.266**	**3**
		4	0.438	
	Public health	0	0.998	
		1	0.710	
		2	0.754	
		**3**	**0.502**	**3**
		4	0.739	
(15) South Korea	Three areas combined	0	1.252	
		**1**	**0.443**	**1**
		2	0.449	
		3	0.505	
		4	0.594	
	Laboratory sciences	0	0.558	
		**1**	**-1.120**	**1**
		2	-0.959	
		3	-0.972	
		4	-0.884	
	Clinical research	**0**	**0.339**	**0**
		1	0.445	
		2	0.386	
		3	0.577	
		4	0.785	
	Public health	0	0.477	
		1	-0.710	
		**2**	**-0.715**	**2**
		3	-0.483	
		4	-0.415	
